# Clinical and Public Health Guidelines: A scholarly home for the science of guidelines

**DOI:** 10.1002/gin2.12007

**Published:** 2024-01-30

**Authors:** Zachary Munn

**Affiliations:** ^1^ The University of Adelaide School of Public Health Ringgold Standard Institution Adelaide Australia

I was not the first person to consider starting a journal dedicated to guidelines. In reality, by the time I presented a proposal formally to the Guidelines International Network (GIN) board in September 2021, the idea to establish a journal (in this digital information age) was far from a novel one. However, it always struck me as odd that although there are tens of thousands of journals with new ones being launched every year, there were no dedicated peer‐reviewed scholarly journals focusing on guideline methods or research, or journals publishing solely guidelines. From my perspective as an evidence synthesiser, implementation scientist and guideline methodologist, this seemed incongruous given there are multiple journals dedicated to systematic reviews (both methods and/or the reviews themselves), health technology assessment and implementation science. While these other fields had their scholarly homes for scientific output, guideline development and methods articles remained scattered in the wind.

GIN is a network of organisations and individuals interested in evidence‐based guidelines and has one of the world's largest international guideline library and registry. Founded in November 2002 and formally incorporated as a company and a Scottish Charity in February 2003, GIN seeks to improve the quality of health care by promoting systematic development of clinical practice guidelines and their application into practice, through supporting international collaboration. With the benefit of hindsight, perhaps an ideal time to have established a journal for the guideline community would have been during the establishment of GIN—although it is fair to say the original founders had more than enough to focus on. As the old saying goes, ‘the best time to plant a tree was 20 years ago, the second best time is now’—and we believe that now, as the guideline field continues to evolve, the soil is ready for the planting.

In November 2021, the GIN board agreed that an expression of interest for a publishing partner should be drafted. During 2022, this was distributed externally and the board sought feedback and input from the broader GIN community, publishers and others interested in the idea of a journal dedicated to guidelines. After considerable feedback and interest, we were proud to announce Wiley as the publisher for this journal. Once an agreement was put in place, we undertook our next task—identifying the inaugural editor in chief for this journal. It was with great pleasure (and some relief after tough competition for the role) that we were able to recruit Dr Ivan Florez to this role. With his enthusiastic nature, passion for guideline methods, comprehensive understanding of the field along with a keen eye for detail, we know he is the perfect candidate for this critical appointment.

We see this dedicated journal for guidelines (and guideline science) having a number of benefits for both GIN as an organisation and also our members and partners. First, an official journal for the network will assist in raising GIN's profile and promoting the work of GIN. The journal will provide an outlet for the working groups and regional communities along with GIN partners and members. Additionally, it will be a vehicle for people wishing to publish their guidelines (or abridged guidelines) as well as the all‐important guideline protocols in the journal. To ensure success the journal will rely on the support of the guideline community, and we aim to provide opportunities for the GIN membership to contribute to the journal in many ways, such as on the editorial board, by acting as peer reviewers and providing content for the journal.

It was a privilege and a pleasure to be invited to write this editorial for the first issue of our journal outlining its history, founding and purpose. Importantly, when saying ‘our’ journal, I refer not only to the GIN board or editorial board of Clinical and Public Health Guidelines, but the entire GIN membership and the broader guideline development and implementation community across the globe. We hope (with time), that this journal will become the scholarly home for the science of guidelines, welcoming a myriad of submissions contributing to and advancing this science in all forms. We look forward to articles on guideline methods work, case studies of guideline projects, exemplar guidelines themselves, implementation considerations and many other types of submissions. As the only international, peer‐reviewed scientific journal dedicated solely to publishing research and methods relating to guidelines as well as guidelines themselves, we hope this journal will have a broad audience for anyone working in health and medical institutions, academic or training organisations or guideline development organisations themselves, including government bodies. We believe the appeal of the journal crosses both academic and clinical populations, and given it will also publish guidelines themselves, will produce impactful work that will assist in achieving GIN's vision of trustworthy and accessible guidance for better health. I invite you to follow closely the progress of our journal as it grows, as I am sure it will be an exciting and interesting time. Additionally, do consider contributing to the journal as well—as we all work together to advance the science of guideline development.

